# Hypocalcemia: A Little Known Cause of Supraventricular Tachyarrhythmia

**DOI:** 10.7759/cureus.38456

**Published:** 2023-05-02

**Authors:** Sualeha Khalid, Isam Albaba, Kristofer Neu

**Affiliations:** 1 Internal Medicine, Stratton VA Medical Center, Albany, USA

**Keywords:** supraventricular tachycardia, qt interval prolongation, hypo-parathyroidism, atrial arrhythmia, s: hypocalcemia

## Abstract

Calcium is an essential electrolyte in impulse generation and contraction of cardiac muscle. Hypocalcemia can occur in cases of parathyroid hormone deficiency, primarily due to inadvertent removal of the parathyroid gland during thyroidectomy, however most cases are idiopathic. We present a case of an adult male who developed sustained narrow complex tachycardia due to hypocalcemia in the setting of untreated idiopathic hypoparathyroidism.

## Introduction

QT interval prolongation is a common electrocardiographic abnormality in hospitalized patients and is known to cause ventricular arrhythmias such as torsade de pointes (Tdp) [1,2]. What is not as well known is that prolonged QT can cause supraventricular tachyarrhythmia (SVT). Medications and electrolyte abnormalities are common causes of acquired long QT syndrome [1]. Common electrolyte abnormalities associated with this are hypokalemia and hypomagnesemia. Hypocalcemia can also prolong the QT and is a rare cause of ventricular arrhythmias. This occurs particularly in childhood and is associated with congenital long QT syndromes [2]. There are a few reported cases of supraventricular arrhythmias in children as well as adults that occurred in association with hypocalcemia [3-5].

## Case presentation

**Figure 1:**
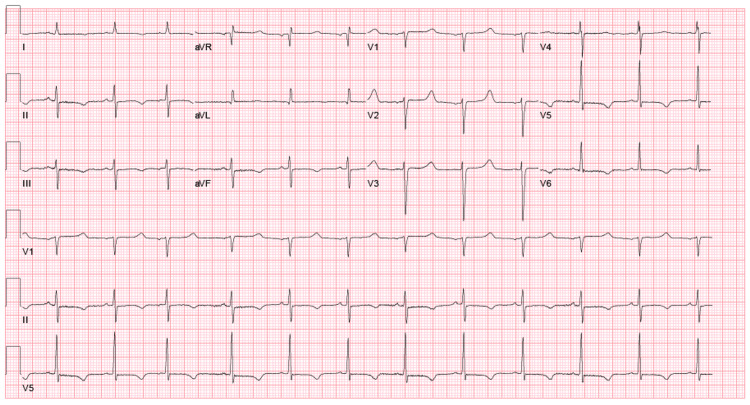
EKG on admission

A 63-year-old man presented to the Emergency Room (ER) with complaints of generalized weakness that started four days prior but denied any other symptoms. His past medical history was significant for hypoparathyroidism that was diagnosed a few years ago after he was found to have low calcium, elevated phosphorus, low parathyroid hormone (PTH), normal vitamin D, and normal 24-hour urine calcium (Table 1). It was thought to be idiopathic in nature as patient had no history of thyroidectomy, personal or family history of autoimmune disease or radiation to neck. He was non-compliant with his calcium supplementation. In the ER, he had a pulse of 60 beats/min and rest of vitals were within normal limits. Physical exam was otherwise unremarkable. Work up demonstrated severe hypocalcemia (4 mg/dL) and hyperphosphatemia (6.8 mg/dL) (Table 1). Electrocardiogram (EKG) showed normal sinus rhythm (Figure 1) with a prolonged QTc of 552 msec. He was admitted for repletion of calcium and further workup. He received intravenous (IV) 2 g calcium gluconate and then continued on slow IV calcium infusion of total of 5 g however continued to have low calcium level (Table 1) the next day with only slight improvement.

**Table 1:** Laboratory Values PTH: parathyroid hormone

Timeline	Lab parameter	Patient Value	Reference range
Clinic Visit	Calcium mg/dL	7.2	8.3-10.6
	Phosphorus mg/dL	4.8	2.5-4.5
	PTH pg/mL	<6.3	14-72
	Vitamin D ng/mL	32.9	21-50
	Urine Calcium mg/24hr	137.8	30-400
Admission	Calcium mg/dL	4	8.3-10.6
	Ionized Calcium mmol/L	0.64	1.15-1.32
	Phosphorus mg/dL	6.8	2.5-4.5
Hospital Day 1	Calcium mg/dL	5.5	8.3-10.6
	Ionized Calcium mmol/L	0.76	1.15-1.32
Hospital Day 2	Calcium mg/dL	6	8.3-10.6
	Ionized Calcium mmol/L	0.8	1.15-1.32
	Phosphorus mg/dL	5.9	2.5-4.5
Hospital Day 4	Calcium mg/dL	8.1	8.3-10.6
	Ionized Calcium	1.11	1.15-1.32

**Figure 2:**
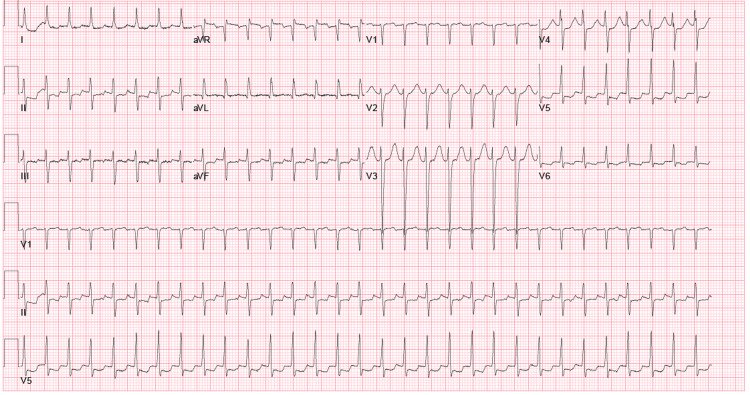
EKG with narrow complex tachycardia

**Figure 3:**
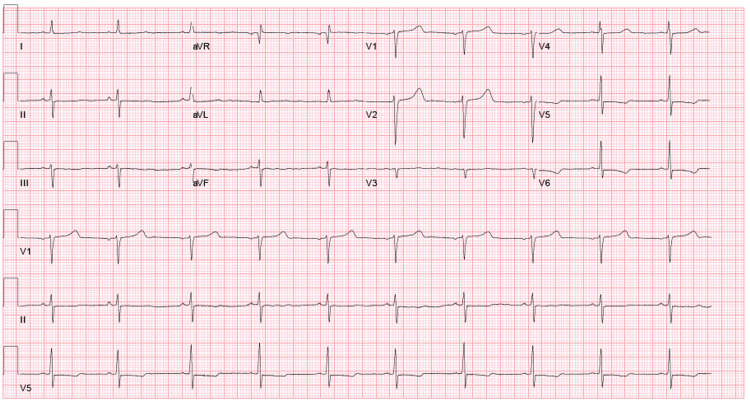
EKG after resolution of narrow complex tachycardia

On day two of hospital stay, the patient developed a narrow complex tachycardia with a heart rate of 170. The patient remained asymptomatic and a 12 lead EKG confirmed a narrow complex tachycardia (Figure 2). The patient received IV adenosine 6 mg then 12 mg, IV metoprolol 5 mg twice, 5 mg IV diltiazem, and IV amiodarone 150 mg with no effect. At this point, IV magnesium 2 g and calcium gluconate 4 g were given, after which patient abruptly converted to sinus rhythm. Figure 3 shows EKG obtained immediately after resolution of arrhythmia.

On review of telemetry from day two, no rhythm abnormalities were noted prior to onset of narrow complex tachycardia. The telemetry rhythm strip and EKG were concerning for atrioventricular nodal re-entry tachycardia (AVNRT) as there were no clear p waves. No continuous anti-arrhythmic or nodal agents were started, and patient was transferred to Intensive Care Unit (ICU) for closer monitoring. Subsequent EKGs in the ICU showed normal sinus rhythm with QTc of 480 msec and lab-work showed low calcium, ionized calcium, elevated phosphorus (Table 1) and no other electrolyte abnormalities. 

To decrease the risk of severe cardiac dysfunction with rapid repletion, he was started on slow infusion of IV calcium with 5 g calcium in 500 cc of normal saline at a rate of 50 cc/hour for a total of 24 hours along with oral calcium carbonate 1 g twice daily. Calcium levels were monitored and improved over the next two days (Table 1). The patient had no repeat arrhythmias and was discharged home on calcium carbonate 1 g twice daily.

## Discussion

Hypocalcemia causes QT prolongation that predisposes to ventricular arrythmia and can lead to development of torsades de pointes. This case demonstrates the occurrence of supraventricular tachyarrhythmia in patients with hypocalcemia. Serum calcium level is tightly regulated by several hormones, one of which is PTH [6]. Deficiency of PTH in most cases is iatrogenic (post-thyroidectomy). Other cases are either idiopathic or a result of autoimmune or infiltrative destruction of the glands. There are also rare acquired and genetic mutations causing hypoparathyroidism [7]. Our patient likely had idiopathic hypoparathyroidism since he did not have history of neck surgery or signs of an infiltrative disease.

**Figure 4:**
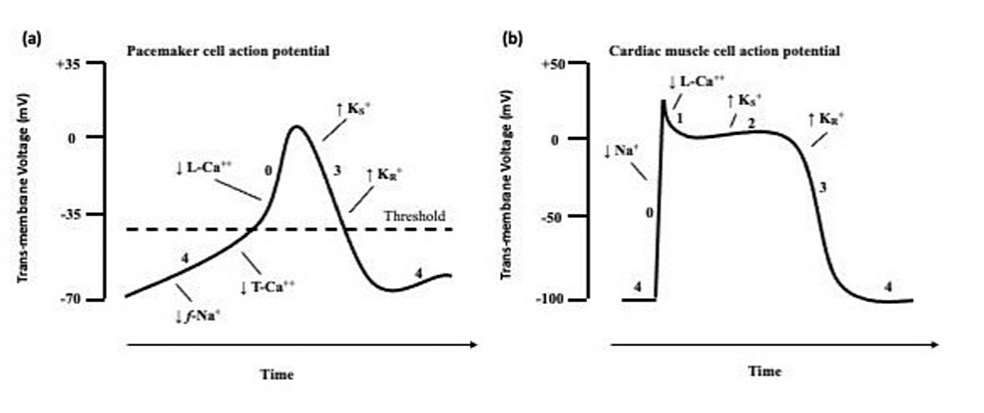
Cardiac Action Potential mV, millivolts; ↓ efflux,  ↑­ influx; *F *-Na^+^, funny sodium channel current, T-Ca^++^, T-type Calcium channels; L-Ca^++^, L-type Calcium channels, K_s_^+^, slow potassium channels, K_r_^+^, rapid potassium channels Image Credit: Isam Albaba

Calcium plays an integral role in the regulation of the action potential (AP) of normal excitable and contractile cardiac tissue (Figure 4). To review, calcium influx through voltage-gated L-type calcium channels causes the rapid depolarization (phase 0) in pacemaker cells. The relatively longer period of influx in contractile tissue and simultaneous potassium efflux results in an isoelectric state (phase 2), giving rise to the flat S-T segment we see on the electrocardiogram. This sustained calcium influx also causes contractility that leads to systole. The closure of these same calcium channels and persistent potassium efflux leads to repolarization (phase 3). Furthermore, besides sodium, calcium influx through T-type channels plays a role in the generation of low-voltage spontaneous depolarization (phase 4), contributing to automaticity of excitable tissue [8].

Atrial tachyarrhythmias develop through one of three major mechanisms [9]: First, increased automaticity results from either enhanced intrinsic automaticity of normal pacemaker tissue (e.g. SA node) or through induced abnormal automaticity of non-pacemaker tissue. Second, the majority of clinically encountered tachyarrhythmias are the result of re-entry. This occurs when a premature impulse travels through a loop consisting of two limbs of differing refractory periods and conduction velocities, resulting in a continuously generating re-entry electrical impulse. Third, triggered activity occurs when a strong enough action potential (AP), called an afterdepolarization occurs within the relative refractory period. Early afterdepolarization (EAD) and delayed afterdepolarization (DAD) occur during phases 3 and 4 of the AP, respectively. Hypocalcemia causes QT prolongation by prolonging phase 2 of the AP. A longer depolarization phase increases the likelihood of early afterdepolarizations (EADs). Triggered activity was the most likely underlying mechanism of supraventricular tachycardia in our patient.

Review of the literature reveals a few cases of hypocalcemia associated with atrial arrhythmias. While underlying causes of hypocalcemia varied and included hypoparathyroidism and rickets, most cases had a prolonged QTc (>500ms) and were treated with IV calcium gluconate [3-5]. In these cases, the type of atrial arrhythmias varied and included atrial fibrillation, atrial flutter, ectopic atrial tachycardia and supraventricular tachycardia. Limited studies on patients with congenital long QT syndrome showed a 10-fold increased risk of atrial tachyarrhythmias in this population compared to the general population [10]. In some of these cases, atrial fibrillation had an f wave pattern with oscillating magnitudes reminiscent of that seen with Tdp. This “atrial torsade de pointes” appearance suggests that the pathogenesis of atrial arrhythmias induced by prolonged QT interval is the same one causing Tdp [11]. One study showed a close association between alterations in AP magnitude and alterations in activity of calcium-activated chloride channels in atrial myocytes, suggesting a potential role of calcium in atrial arrhythmias [12].

The narrow complex nature of the tachyarrhythmia in our case confirms an atrial origin. There were no discernible P waves, nor were there flutter waves, ruling out both atrial fibrillation and flutter, respectively. The onset and termination of the arrhythmia were both abrupt, which is a classic feature of atrioventricular re-entry tachycardia (AVRT) and atrioventricular nodal re-entry tachycardia (AVNRT). Atrial tachyarrhythmias are common, and it is possible this arrhythmia was not secondary to hypocalcemia. However, the multiple reported cases that illustrate an association between hypocalcemia and atrial arrhythmia suggest our theory may be true. Additionally, there was lack of response to beta blockers and calcium channel blockers. This might be because nodal agents will not be effective if the primary abnormality (hypocalcemia) that predisposes to tachyarrhythmia persists. The patient’s arrhythmia only responded after repletion of calcium, which likely stabilized the cardiac membrane potential and subsequently aborted the supraventricular arrhythmia.

## Conclusions

Contractile and excitable cardiac tissues rely on the homeostasis of electrolytes in the body, especially calcium. Derangements in calcium levels alter the initiation and duration of action potential, increase the risk of after depolarizations and hence, increase risk of cardiac arrhythmias. The risk of ventricular arrhythmia in patients with hypocalcemia is well known. Atrial arrhythmias are less commonly studied with hypocalcaemia and further research focused on the characterization of arrhythmias in the hypocalcaemia population would be helpful to examine a possible association between hypocalcaemia and atrial tachyarrhythmia.
